# Supplementary material for: Targeted methods for epigenetic age predictions in mice
Source: Sci Rep. 2020 Dec 31;10:22439. doi: 10.1038/s41598-020-79509-2 (PMC7775437; doi:10.1038/s41598-020-79509-2)
Supplement: Supplementary file 1 — Supplementary Information. [file 41598_2020_79509_MOESM1_ESM.pdf]

Supplementary Material

Targeted methods for epigenetic age predictions in mice

Yang Han, Miloš Nikolić, Michael Gobs, Julia Franzen, Gerald de Haan, Hartmut Geiger, and Wolfgang Wagner

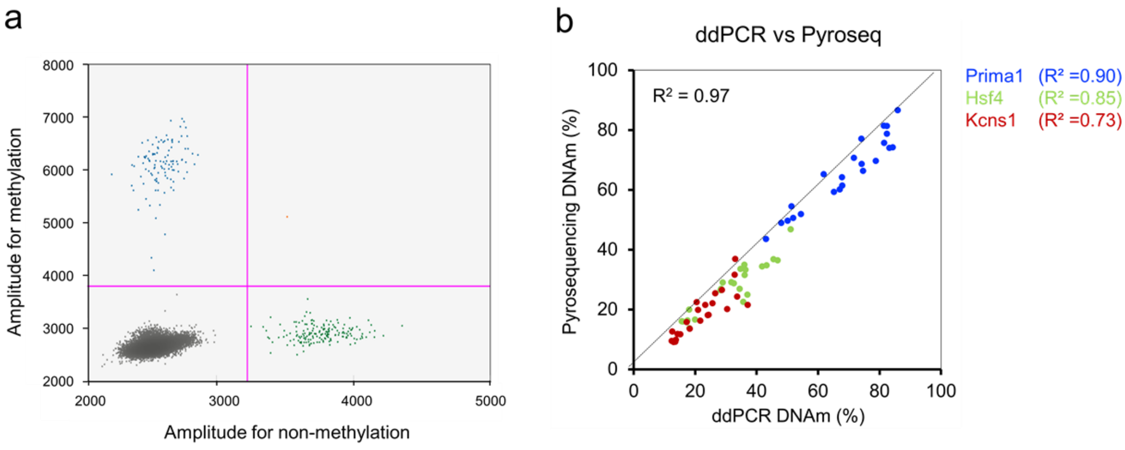

Supplementary Fig. 1. Comparative analysis of age-associated DNAm by ddPCR and Pyrosequencing.
a)This plot exemplarily depicts ddPCR results of DNAm in *Prima1* (Blue: positive droplets for methylated; Green: positive droplets for unmethylated; Orange: double positive droplets; Grey: negative droplets). **b)** DNAm analysis at three age-associated regions were compared in 23 samples with ddPCR and pyrosequencing. Overall, the DNAm levels correlated very good between the two methods.

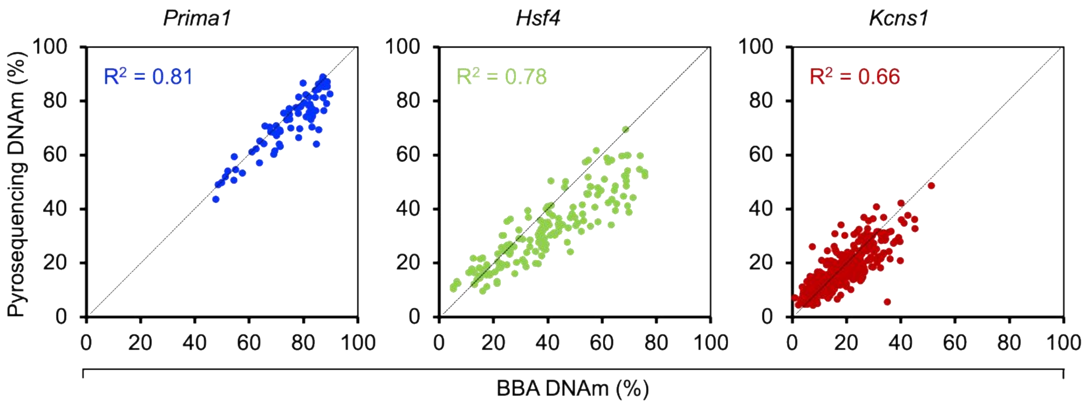

Supplementary Fig. 2. Comparison of DNAm levels in pyrosequencing and BBA-seq.
The DNAm levels at the three age-associated genomic regions were compared in 23 blood samples with barcoded bisulfite amplicon sequencing (BBA-seq) and pyrosequencing. All CpGs that were covered by both types of measurements were included into the comparison.

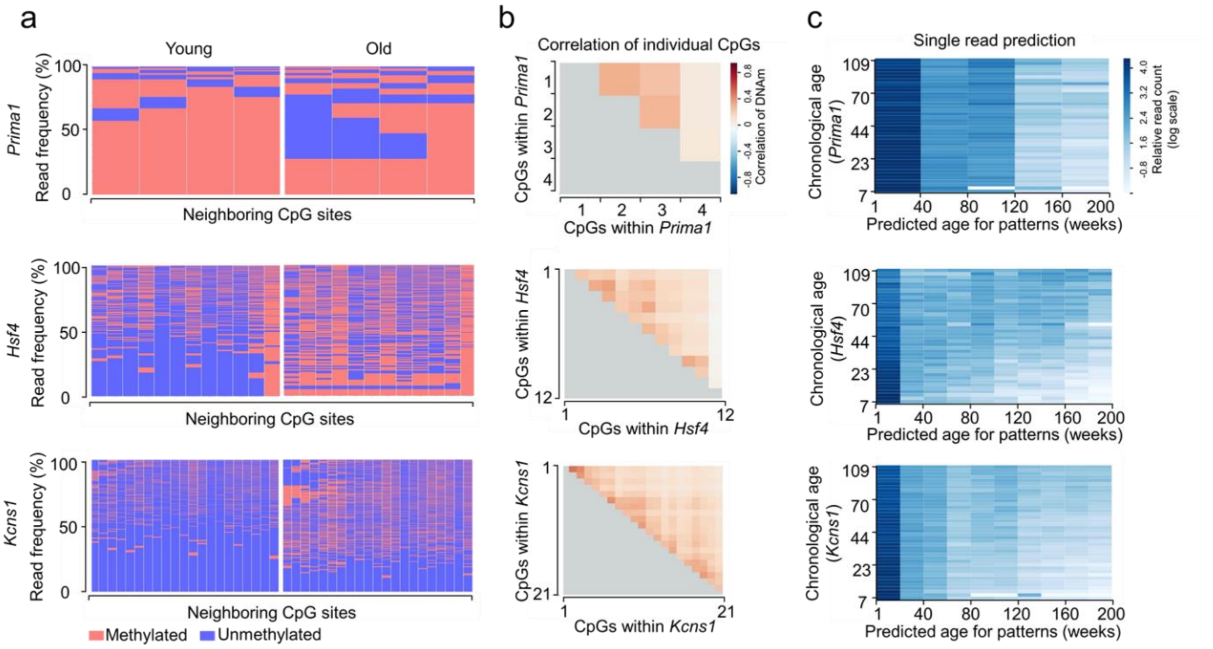

Supplementary Fig. 3. Analysis of age-associated DNAm patterns within individual BBA-seq reads in C57BL/6 mice of the validation sets.

a) Heatmaps display the frequency of DNAm patterns within *Prima1*, *Hsf4* and *Kcns1* amplicons from BBA-seq data. One young (7 weeks) and one old (109 weeks) C57BL/6 mouse samples from BBA-seq validation sets were compared. **b)** Pearson correlation of DNAm among neighboring CpGs within three amplicons from BBA-seq validation sets. **c** According to single BBA-seq read prediction of the training set, epigenetic ages were estimated based on the binary sequel of methylated and non-methylated CpGs for three amplicons separately. Each plot classified relative read count of each donor in the two validation sets for predicted ages between 0 and 200 weeks (Relative read count was normalized by logarithmic scale of read count per sample).

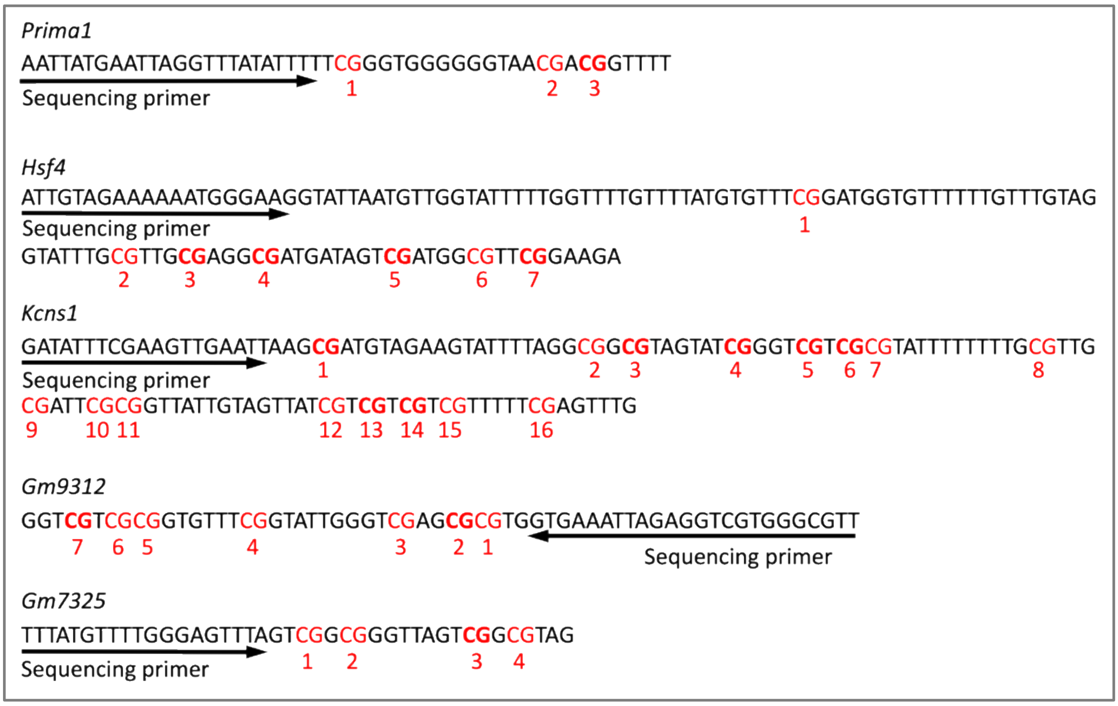

Supplementary Fig. 4. Targeted sequences of pyrosequencing assays.
Sequences for the five genomic regions (after bisulfite conversion). The CpG sites are depicted in red and numbered consecutively by their dispensation order. The relevant 15 CpGs selected by the machine learning model are highlighted in bold.

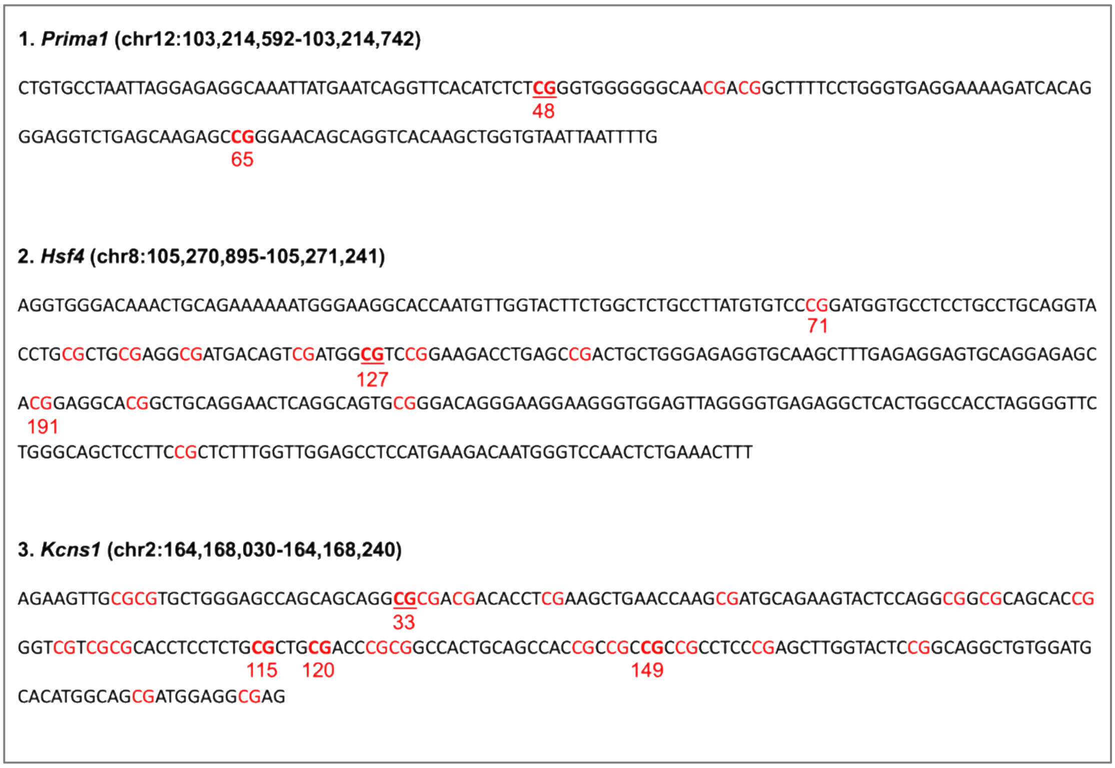

Supplementary Fig. 5. Targeted sequences for BBA-seq.
Sequences for three genomic regions that were analyzed by BBA-seq are depicted and all CpG sites are highlighted in red. The age-related CpGs selected by machine learning approach are highlighted in bold and by their ordering number in the sequences (underlined CpG were used in the multivariable linear model).

Supplemental Table 1. Advantages and limitations of different targeted approaches.

	Pyrosequencing	ddPCR	BBA-seq
Consumables per sample (including bisulfite conversion) *	~ 25 €	~ 20 €	~ 40 € ***
Working time **	10 h	10 h	14 h
Time until results	2-3 days	2-3 days	5-7 days
Preferred DNA amount for bisulfite conversion	200 - 500 ng	200 - 500 ng	200 - 500 ng
Minimum input quantity of converted DNA	30 - 60 ng (10-20 ng per reaction)	30-60 ng (10-20 ng per reaction)	30-60 ng (10-20 ng per reaction)
Requirement of bioinformatic skills	low	low	high
Maximum read length	~ 100 bp	1 CpG	~ 500 bp

* Please note that the costs for consumables may vary considerably between suppliers, country, and institutional arrangements. The projected costs are only for orientation.
** The working time needs to be taken into account for cost calculation. However, this is largely dependent on the number of samples that can be processed in parallel.
*** Particularly for BBA-seq the costs for consumables are also dependent on the number of samples that can be analyzed on the same sequencing lane. They may decrease significantly with higher sample numbers.

Supplementary Table 2. Primers for pyrosequencing assays.

Primer	Sequence
Prima1	
Forward	5'-TTGTGTTTAATTAGGAGAGGTAAATTATGAATTAGGTTTATA-3'
Reverse	5'-Biotin-CAAAATTAATTACACCAACTTATAACCTACTATTC-3'
Sequencing	5'-AATTATGAATTAGGTTTATATTT-3'
Hsf4	
Forward	5'-GTGAGTAGTAAGGTGGGATAAATTGTAGAAAAAATG-3'
Reverse	5'-Biotin-TCCCTACTCTCCTACACTCCTCTCAAAACTTA-3'
Sequencing	5'-ATTGTAGAAAAAATGGGAA-3'
Kcns1	
Forward	5'-GGTTGAGAGGGTGGTAGAAGAAGTTG-3'
Reverse	5'-Biotin-ACTCCCCTCCATCCCTACCATATACATCCA-3'
Sequencing	5'-GAAGATATTTAGAAGTTGAATT-3'
Gm9312	
Forward	5'-Biotin-TTGTTTTGGGGTATTAGAAATTTTTTT-3'
Reverse	5'-CCTAACCATACTAAACCAAATCTCTATATCTAAAT-3'
Sequencing	5'-AACCCCCACCACCTCTAATTTCAC-3'
Gm7325	
Forward	5'-TGTTGGTTGAGGATAAAGAGTAGATAGTTTAGTAGAGT-3'
Reverse	5'-Biotin-TTCCCTTTACAAATACAAATCCTACCATA-3'
Sequencing	5'-TTTATGTTTTGGGAGTTTA-3'

Supplementary Table 3. Machine learning model based on 15 CpGs (Pyrosequencing)

Gene name	Chromosome	CpG#	Position	R^2	Coefficients
(Intercept)					-1.62345
Prima1	chr12	3	103214656	0.62	-0.29869
Hsf4	chr8	3	105271000	0.95	1.254757
Hsf4	chr8	4	105271006	0.91	0.327821
Hsf4	chr8	5	105271015	0.90	0.467297
Hsf4	chr8	7	105271025	0.81	0.001369
Kcns1	chr2	1	164168088	0.81	0.170725
Kcns1	chr2	3	164168113	0.84	0.089469
Kcns1	chr2	4	164168121	0.74	0.320572
Kcns1	chr2	5	164168126	0.73	0.343513
Kcns1	chr2	6	164168129	0.79	0.05701
Kcns1	chr2	13	164168175	0.82	0.326642
Kcns1	chr2	14	164168178	0.81	0.307202
Gm9312	chr12	2	24252014	0.47	0.055652
Gm9312	chr12	7	24252050	0.25	0.434457
Gm7325	chr17	3	45601568	0.37	-0.13861

This model was trained with a machine learning approach (linear model with L1 regularization; lambda = 1.570242), as described before (Han et al., 2018). CpG# indicates the relevant age-related CpGs of the amplicon, as also depicted in Supplementary Fig. 4.

Supplementary Table 4. Primers for ddPCR assays.

Primer	Sequence
Prima1	
Forward	5'- GGAGAGGTAAATTATGAATTAGG -3'
Reverse	5'- ACTCTTACTCAAACCTCCCT -3'
Probe	6-Fam - TATATTTTTCGGGTGGGGG -BHQ-1[a]
	Hex- TATATTTTTTGGGTGGGGG -BHQ-1[b]
Hsf4	
Forward	5'- AATGTTGGTATTTTTGGTTTTGTTT -3'
Reverse	5'- AAAACTTACACCTCTCCCAACAAT -3'
Probe	6-Fam - TGTGTTTCGGATGGTGTTTTTTGT - BHQ-1[a]
	Hex - TGTGTTTTGGATGGTGTTTTTTGT - BHQ-1[b]
Kcns1	
Forward	5'- TTGGGAGTTAGTAGTAGGYG -3'
Reverse	5'- ATACATCCACAACCTACCRA -3'
Probe	6-Fam - AGTTGAATTAAGCGATGTAGAAGTATTTTA - BHQ-1[a]
	Hex - AGTTGAATTAAGTGATGTAGAAGTATTTTA - BHQ-1[b]

[a] Probe targeting the methylated sequence.
[b] Probe targeting the non-methylated sequence.

Supplementary Table 5. Multivariable model for ddPCR.

Gene Name	CHR	Map Info	Coefficients	P value
(Intercept)			-11.56	0.83
Prima1	12	103214640	-0.26	0.60
Hsf4	8	105270966	2.33	0.001
Kcns1	2	164168063	0.43	0.63

Supplementary Table 6. Primers for BBA-seq assays.

Primer	Sequence
Prima1	
Forward	5'- CTCTTTCCCTACACGACGCTCTTCCGATCTTTGTGTTTAATTAGGAGAGGTAAATTATGAATTAGGTTTATA -3'
Reverse	5'- CTGGAGTTCAGACGTGTGCTCTTCCGATCTCAAAATTAATTACACCAACTTATAACCTACTATTC -3'
Hsf4	
Forward	5'- CTCTTTCCCTACACGACGCTCTTCCGATCTAGGTGGGATAAATTGTAGAAAAAATG -3'
Reverse	5'- CTGGAGTTCAGACGTGTGCTCTTCCGATCTAAAATTTCAAAATTAAACCCATTATCTTCA -3'
Kcns1	
Forward	5'- CTCTTTCCCTACACGACGCTCTTCCGATCTAGAAGTTGYGYGTGTTGGGAGT -3'
Reverse	5'- CTGGAGTTCAGACGTGTGCTCTTCCGATCTCTCRCCTCCATCRCTACCATATAC -3'

Supplementary Table 7. Multivariable model for BBA-seq.

Gene Name	CHR	Map Info	Coefficients	P value
(Intercept)			11.92	0.74
Prima1.48	12	103214640	-0.53	0.15
Hsf4.127	8	105271022	1.08	0.03
Kcns1.33	2	164168063	1.53	0.003

The numbers at the target sites indicate the relevant age-related CpGs of the amplicon, as also depicted in Supplementary Fig. 5.

Supplementary Table 8. Machine learning model by Lasso (BBA-seq)

	Target sites*	CHR	Map Info	Coefficients
	(Intercept)			5.18
1	*Prima1*.65	12	103214657	-0.26
2	*Hsf4*.71	8	105270966	0.83
3	*Hsf4*.191	8	105271086	0.11
4	*Kcns1*.33	2	164168063	0.19
5	*Kcns1*.115	2	164168145	0.41
6	*Kcns1*.120	2	164168150	0.52
7	*Kcns1*.149	2	164168179	0.43

This model was trained with a machine learning approach, although the number of samples may not be enough for cross-validation on the data. The model should therefore be retained when a larger set of samples is available. The numbers at the target sites indicate the relevant age-related CpGs of the amplicon, as also depicted in Supplementary Fig. 5.
